# Uneven terrain exacerbates the deficits of a passive prosthesis in the regulation of whole body angular momentum in individuals with a unilateral transtibial amputation

**DOI:** 10.1186/s12984-019-0497-9

**Published:** 2019-02-04

**Authors:** Jenny A. Kent, Kota Z. Takahashi, Nicholas Stergiou

**Affiliations:** 1 Department of Biomechanics and Center for Research in Human Movement Variability, College of Education, University of Nebraska at Omaha, 6160 University Drive South, Omaha, NE 68182-0860 USA; 2 College of Public Health, 984355 University of Nebraska Medical Center, Omaha, NE 68198-4355 USA

**Keywords:** Gait, Biomechanics, Amputees, Passive prostheses, Uneven terrain, Motor learning, Angular momentum

## Abstract

**Background:**

Uneven ground is a frequently encountered, yet little-studied challenge for individuals with amputation. The absence of control at the prosthetic ankle to facilitate correction for surface inconsistencies, and diminished sensory input from the extremity, add unpredictability to an already complex control problem, and leave limited means to produce appropriate corrective responses in a timely manner. Whole body angular momentum, *L,* and its variability across several strides may provide insight into the extent to which an individual can regulate their movement in such a context. The aim of this study was to explore *L* in individuals with a transtibial amputation, when challenged by an uneven surface. We hypothesized that, similar to previous studies, sagittal plane *L* would be asymmetrical on uneven terrain, and further, that uneven terrain would evoke a greater variability in *L* from stride to stride in individuals with amputation in comparison to unimpaired individuals, due to a limited ability to discern and correct for changing contours beneath the prosthetic foot.

**Methods:**

We examined sagittal plane *L* in ten individuals with a unilateral transtibial amputation and age- and gender- matched control participants walking on flat (FT) and uneven (UT) treadmills. The average range of *L* in the first 50% of the gait cycle (*L*_R_), the average *L* at foot contact (*L*_C_) and their standard deviations (v*L*_R_, v*L*_C_) were computed over 60 strides on each treadmill.

**Results:**

On both surfaces we observed a higher *L*_R_ on the prosthetic side and a reduced *L*_C_ on the sound side (*p* < 0.001) in the amputee cohort, consistent with previous findings. UT invoked an increase in *L*_C_ (*p* = 0.006), but not *L*_R_ (*p* = 0.491). v*L*_R_, and v*L*_C_ were higher in individuals with amputation (*p* < 0.001, *p* = 0.002), and increased in both groups on UT (p < 0.001).

**Conclusions:**

These findings support previous assertions that individuals with amputation regulate *L* less effectively, and suggest that the deficits of the prosthesis are exacerbated on uneven terrain, potentially to the detriment of balance. Further, the results indicate that a greater demand may be placed on the unaffected side to control movement.

**Electronic supplementary material:**

The online version of this article (10.1186/s12984-019-0497-9) contains supplementary material, which is available to authorized users.

## Background

The ability to walk confidently on non-level ground is vital when any activity to be pursued involves ambulation outside a home or institutional environment. Uneven terrain poses a challenge for people with lower limb amputation [1], can lead to activity avoidance [2], and can increase fall risk [3].

Walking on non-level ground, by nature, demands subtle or marked alterations to movement on a step-by-step basis, in order to maintain balance and propulsion in the face of inconsistencies underfoot. For individuals with a transtibial amputation, appropriate changes must be made lacking the precise control, active propulsion and adaptable compliance of a natural ankle, alongside the tactile and proprioceptive mechanisms that aid in the determination of the quality and contour of the ground [4].

**Fig. 1:**
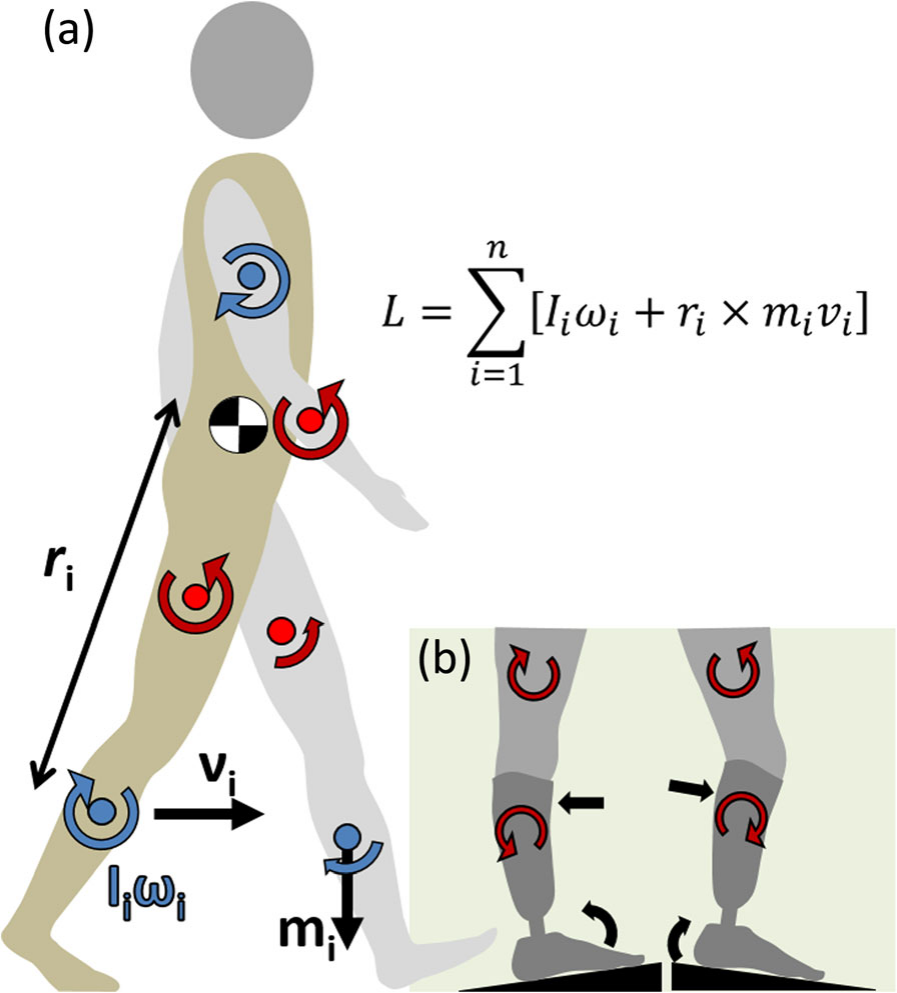
Sagittal plane rotational dynamics. (**a**) Whole body angular momentum, *L*, as the summed momenta of the individual segments, *i*, illustrated in the sagittal plane (**b**) Influence of uneven ground on passive prosthesis motion and segmental rotations

In order to maintain balance in addition to moving the body forward, locomotion requires the effective coordination of rotations of multiple segments of the body (e.g. foot, thigh, trunk) about the joints [5], given the external forces presented by the task and environmental context. In normal, level walking, the momenta of the individual segments have been shown to largely cancel out, such that the angular momentum of the whole body, *L*, i.e. the total rotational momentum of all segments combined acting about their resultant center of mass (Fig. 1a), is close to zero [6]. The generation and manipulation of *L* during locomotion are largely controlled through muscle activity under the influence of external moments induced via the interaction of the body with the ground [5, 7]. Large deviations in *L*, of either external or internal origin, may disrupt balance during locomotion (see e.g. [8]). Importantly, an inability to rapidly and effectively control *L* has been linked to an increase risk of falls [9].

Physically, the time rate of change of *L* represents the sum of the external moments acting on the body about the center of mass [5]. Any alterations of the magnitude or direction of the ground reaction force that occur when walking on uneven ground will therefore have an effect on *L*. Consequently, muscle activity patterns must be adapted appropriately to regulate it. As such, *L* provides a measure based on whole body dynamics that may provide insight into the extent to which an individual can effectively re-orchestrate the rotations of body segments given changing balance demands [10], pertinent to fall risk [9].

The multiple articulations of the natural foot and ankle complex aid the accommodation of changes in terrain [11]. To cope with changing demand, the posture, rigidity and mechanical behavior of a natural foot and ankle may be modified during walking, enabling action to be fit to context [12]. Through biomechanical modelling, for example, it has been shown that the ankle plantarflexors play a primary role in sagittal plane control of *L* both during early and late stance [5]. It has been shown that modulation of lower limb muscle activity occurs rapidly, and with even very modest changes (less than 3°) in surface incline [13].

In contrast, the behavior of a prosthetic lower extremity is not under direct control of the user, and will depend on several factors related to the design of the component alongside the manner with which it is utilized [14]. The majority of ankle components prescribed to users who have, or may be able to attain, the ability to tackle uneven ground in their daily life are passive devices. These designs have no controllable articulation at the ankle; instead incorporating cantilever-like keels that deflect under load, acting as a fixed spring that stores and returns energy at specific points in the gait cycle [15, 16]. The inclusion of various design features, such as a split toe and heel to increase medial-lateral compliance; a rotation unit to permit axial rotation; and a vertical shock absorbing pylon, may aid the user in walking on uneven surfaces. Essentially, however, the action of such a foot under different loading conditions, and subsequent nature of the load delivered to the body, will be largely determined by device design, geometry and material properties [17], and is not user-directed.

Individuals with amputation show a greater range of *L* in the sagittal plane during level walking [18, 19], and a smaller reduction in *L* on declined slopes in comparison to able-bodied individuals, implying less effective regulation [18–20]. The potential for destabilization may be exacerbated on a surface that continually fluctuates as regulation will require an appropriate re-orchestration of movement on a step-by-step basis.

It is at present unknown how effectively individuals with amputation walking with passive prostheses orchestrate their movement on uneven ground, and the extent to which the residual limb that relies on a prosthetic foot for contact, support and propulsion contributes to the regulation of *L*. We postulate that peaks and dips in the terrain surface induce changes in external moments during prosthetic stance phase (Fig. 1b) due to the fixed spring constant, leading to inappropriate coordinative movement strategies and destabilizing interactions as the sound swing limb contacts the ground.

The aim of this study was to explore the extent to which *L* is effectively regulated by individuals with a transtibial amputation wearing passive devices, when challenged by an uneven surface. We hypothesized that, similar to previously reported findings in level walking [18, 19, 21], sagittal plane *L* would be asymmetrical on uneven terrain due to the deficits of the prosthetic limb: on average a greater range of *L* would occur during the prosthetic limb single stance phase due to a reduction in prosthetic limb braking capacity [21], and a lower magnitude of *L* would be observed at sound side foot contact due to a prosthetic side propulsion deficit during late stance of the affected side [21]. In conjunction, we anticipated that the effect of the uneven terrain would be exacerbated in individuals with amputation due to the inability to effectively coordinate movement in light of functional and sensory deficits.

We further hypothesized that uneven terrain would evoke a greater variability in sagittal plane *L* from stride to stride in individuals with amputation in comparison to able-bodied control participants. More specifically, (i) *L* would be more variable at foot contact of the sound limb, as a reflection of a lack of control of angular momentum in the preceding prosthetic stance phase, and (ii) the range of *L* would be more variable during prosthetic stance phase in comparison to sound stance phase due to the lack of ability to adequately discern and correct for changing contours beneath the prosthetic foot.

## Methods

### Participants

All procedures were approved by the University of Nebraska Medical Center and the VA Nebraska-Western Iowa Health Care System Institutional Review Boards. Eleven individuals with a transtibial amputation were recruited from local and VA prosthetics clinics and provided written consent to participate. One participant was later excluded due to a notably slow self-selected walking speed during treadmill walking that resulted in gait deviations that were observably different from over ground walking on the laboratory floor. All participants were experienced prosthesis users, had no neurological disease or impairment that might affect gait other than diabetes, and were able to walk with a prostheses independently, with no walking aids (Table 1). All participants had suction or pin-lock suspension (6 and 3 of 10 participants respectively), with the exception of one individual who used a vacuum socket (Table 1, participant 4) and wore energy storage and return-type foot components classified as higher activity devices (Medicare level K3 or above; Table 1). The components varied by design, with 4 incorporating a split toe and/or heel, 2 with torsion adapters and 3 incorporating a vertical shock-absorption feature. The participants were age- and gender- matched with unimpaired individuals recruited from the student and staff body of UNO and the local community. Independent samples t-tests indicated that the unimpaired individuals were on average shorter and lighter (*p* = 0.035 and *p* = 0.032 respectively; Table 1).

**Table 1:** Study cohort

			Group								
			Amputation						*No impairment*		
#		Side	Etiology	Age (yrs)	Height (m)	Mass (kg)	Yrs Amp	Prosthetic foot (Company)	*Age* *(yrs)*	*Height (m)*	*Mass (kg)*
1	M	R	Diabetes	65	1.78	109.8	5	Reflex Rotate (Ossur)	*64*	*1.77*	*89.8*
2	F	R	Arthritis	70	1.66	100.7	8	Panthera (Medi)	*73*	*1.56*	*78.5*
3	M	R	Trauma	64	1.90	109.3	10	Duralite (Ohio Willow Wood)	*60*	*1.66*	*90.7*
4	M	R	Trauma	57	1.80	112.9	5	Thrive (Freedom Innovations)	*59*	*1.81*	*74.0*
5	M	R	Trauma	68	1.80	118.8	9	Rush 87 (Ability Dynamics)	*62*	*1.70*	*104.2*
6	M	R	Trauma	69	1.87	99.7	12	Rush 87 (Ability Dynamics)	*73*	*1.71*	*88.2*
7	M	L	Diabetes	53	1.83	102.5	7	Variflex (Ossur)	*55*	*1.83*	*10.7*
8	M	L	Congenital	33	1.78	93.4	32	Rush 87 (Ability Dynamics)	*32*	*1.76*	*68.0*
9	M	L	Trauma	49	1.80	83.5	7	Silhouette VS (Freedom Innovations)	*47*	*1.76*	*131.1*
10	F	L	Trauma	37	1.70	66.2	14	Rush 87 (Ability Dynamics)	*36*	*1.66*	*59.0*
Mean				56.5	1.79	99.7	10.7		*56.1*	*1.72*	*79.4*
SD				13.3	0.07	15.5	8.0		*14.0*	*0.08*	*31.4*

Ten experienced transtibial prosthesis users age- and gender- matched to unimpaired individuals (in italics)

### Procedures

Data were collected in a university biomechanics laboratory setting. Participants wore their own footwear and prosthesis (where applicable), a tight fitting athletic suit for the purpose of motion capture, and a ceiling-mounted harness for all trials. Passive retro-reflective markers were placed on the shoes, legs (and prosthesis), pelvis, trunk and hands (see Additional file 1 for full description).

**Fig. 2:**
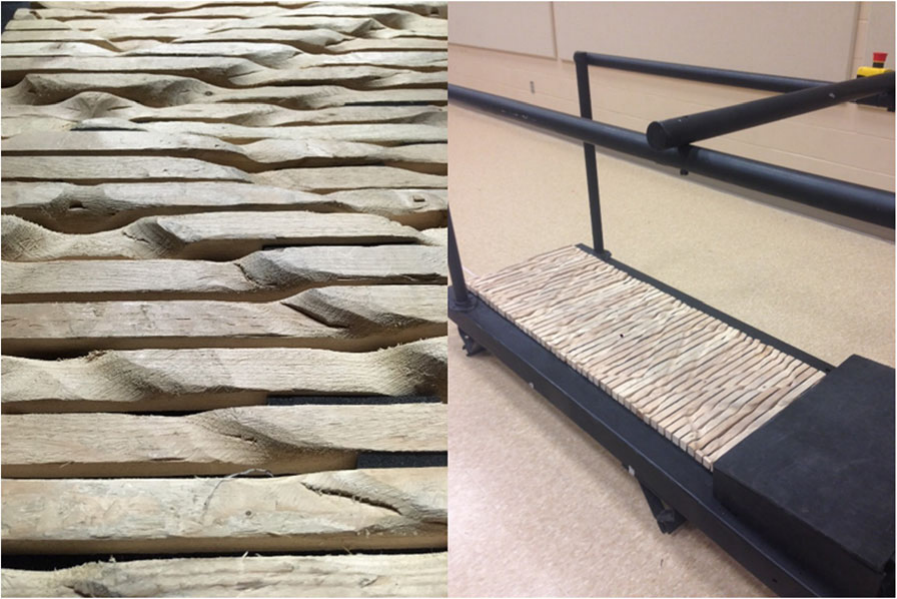
Uneven terrain treadmill. Manually shaped wooden slats were affixed to the belt of a standard treadmill to provide the uneven surface

Walking was captured on two different treadmill surfaces - flat (FT); Tandem Treadmill, AMTI, Inc., Watertown, MA, USA) and uneven (UT); on an uneven terrain treadmill. The UT treadmill utilized was an in-house custom-built device (Fig. 2) with a walking surface comprised of 107 wooden slats, manually shaped to form a repeating pattern that is reflected and offset to give both feet an equal probability of encountering the same contours. The pattern was designed to promote a slightly different interaction on each step unless targeting is attempted, but sufficiently shallow to permit heel-toe gait (4 levels: 0 mm, 7 mm, 14 mm and 22 mm). The device has been previously shown to successfully invoke different midstance postures at each step, thus perpetually changing the direction of action of the ground reaction force during walking [22].

Participants initially walked for 2–3 min on each treadmill to determine the walking speed that would be used for both trials. On the FT treadmill first, participants began walking with their hands lightly touching the handrails if necessary, and were asked to progress to removing their hands from the rails if possible, when comfortable. The treadmill speed was progressively incremented until the participant stated that a comfortable speed has been reached. It was then increased until the participant stated that it was ‘too fast’, then decreased until ‘comfortable’ again. This speed was maintained for a minute, at the end of which the participant was asked to confirm that they were satisfied with it. The process was then repeated on the UT treadmill. This procedure served two purposes. First, it allowed us to determine if there were any large differences in preferred speed that might lead to a demand for uncomfortably slow or fast walking in one or the other of the trials. Second, it enabled participants to familiarize themselves with walking on both treadmills. Differences between self-selected walking speeds in the two terrain conditions were non-significant (mean (SD): FT = 1.1 (0.2) m/s; UT = 1.0 (0.2) m/s, *p* = 0.07) and in order to maintain consistency across terrain conditions, the speed that was selected during the uneven terrain treadmill familiarization was used for both test trials. Participants walked for two minutes at their set walking speed on FT, and then on UT after a rest period lasting at least two minutes. Order of testing was not randomized to avoid potential carry over effects from walking on the uneven terrain. Kinematic data were captured at 100 Hz using a 12-camera optical motion capture system (Raptor, Motion Analysis Corporation, Santa Rosa, CA, USA).

### Data processing

Kinematic data were tracked in Cortex software (Motion Analysis Corp., Santa Rosa, CA, USA) and exported to Visual 3D (C-motion, Germantown, MD, USA) for further processing. A model consisting of 10 segments (lower limb, pelvis, trunk and hands), based on Calibrated Anatomical Systems Technique [23], was applied (see Additional file 1). Marker position data were filtered using a 7 Hz 4th order low pass Butterworth filter, determined from preliminary data using power spectral analysis as described by Winter [24, 25]. The filtered preliminary data were visually inspected against raw data trajectories to confirm that attenuation due to filtering would be negligible. Foot contact events (foot on, foot off) were estimated using a kinematic algorithm [26] and visually inspected for erroneously identified occurrences.

Lower limb segment positions and velocities were computed in Visual3D. The center of mass of the body was approximated from segment positions and estimated segment masses of the feet, lower legs, thighs, pelvis and trunk using inertial values from Hanavan et al. [27]. Based on the general model of Ferris et al. [28], the segment mass of the prosthetic shank was reduced from 4.65 to 3.3% body mass, the center of mass position to 21% of the segment length and the moment of inertia about an axis through the origin parallel to the flexion-extension axis of the limb to 17% of the segment length. Although an approximation, this generalization has been shown to give good approximations of more stringently measured, individualized, inertial parameters [28].

*L* was calculated as the summation of the angular momenta of each segment within the model about the body center of mass in the sagittal plane [6, 29]:

$$ L=\sum \limits_{i=1}^n\left[{I}_i{\omega}_i+{r}_i\times {m}_i{v}_i\right] $$

where, for each segment i, I_i_ is the individual moment of inertia based on the designated geometrical approximation [27], ω_i_ is angular velocity, r_i_ is the distance from the center of mass to the whole body center of mass, m_i_ is the mass and v_i_ the linear (translational) velocity (Fig. 1a). *L* was calculated and reported within the global coordinate system [6, 29], i.e. about the horizontal axis orthogonal to the direction of progression of the treadmill belt. Values were normalized to body mass, height and walking speed to facilitate comparisons across individuals and with previously reported values [18, 19].

The first 60 strides of walking within which complete marker data were available were selected for analysis. For all but one control participant this was the first 60 strides consecutively. Two angular momentum measures were calculated bilaterally within each walking trial. First, to capture the condition the new stance limb accepts on loading, *L* at the estimated instance of foot contact (*L*_c_) was extracted. As peak values of *L* in the sagittal plane tend to follow the instance at which the foot contacts the ground it may be this time point at which balance requires the greatest control [30], dynamic balance is at its most vulnerable, and the sound limb encounters its greatest control challenge. The range, i.e. maximum minus minimum value, of *L* during the first 50% of the gait cycle (*L*_R_) was calculated as a measure of *L* regulation [18, 22]. This corresponds to the period between ipsilateral foot contact and approximate contralateral foot contact, and incorporates the peak value following foot contact and the minimum at midstance. The average values of *L*_C_ and *L*_R_ and their standard deviations (v*L*_C_ and v*L*_R_) were calculated to test our first and second hypotheses respectively.

### Statistical analysis

For each control participant, the ‘prosthetic’ side was selected to be consistent with the amputated side of the matched participant, essentially randomizing the side. This resulted in 4 left and 6 right ‘prosthetic’ sides. Mixed 3-factor ANOVAs with repeated measures factors of limb (2 levels; sound and prosthetic) and terrain (2 levels; FT and UT), and between-subjects factor of group (2 levels; amputation and no impairment), were used to test our first hypothesis, i.e. that *L* would be asymmetrical on uneven terrain using the variables *L*_c_ and *L*_R_, and our second hypothesis, i.e. that uneven terrain would evoke a greater variability in *L*, using the variables v*L*_C_ and v*L*_R_. Post hoc pairwise comparisons with Bonferroni correction were performed when significant differences were identified. Significance for all comparisons was set at 0.05. Partial eta squared (η_P_^2^) was calculated as a measure of effect size. Eta squared (η^2^) measures the proportion of the total variance in a dependent variable that is associated with the membership of different groups defined by an independent variable, with values of 0.01, 0.06 and 0.14 considered small, medium and large effect sizes, respectively [31]. η_P_^2^ is a similar measure in which the effects of other independent variables and interactions are partialled out.

## Results

### Average (mean) sagittal plane whole body angular momentum at foot contact (*L*_C_) and during stance phase (*L*_R_) (hypothesis 1)

**Fig. 3:**
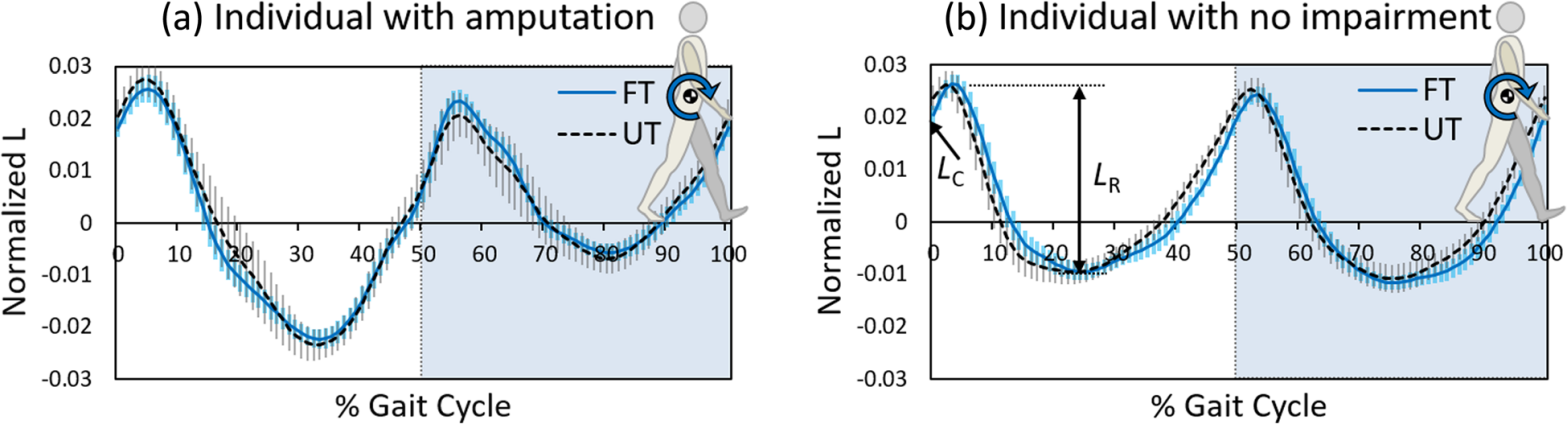
Whole body sagittal plane angular momentum, *L*. Values normalized to mass, height and speed, and time normalized to the right stride (dimensionless). Values are positive in the clockwise direction. Individual participant profiles: (**a**) prosthetic side gait cycle of 65 yr. old male with right unilateral amputation (**b**) matched prosthetic side gait cycle of 64 yr. old male with no amputation. Mean of 60 strides ±1 standard deviation. FT – flat terrain, UT – Uneven terrain. *L*_C_ – Value of *L* at foot contact; *L*_R_ – Range of *L* over first 50% of the gait cycle (within non-shaded region)

As anticipated, and consistent with previous findings [18, 19], sagittal plane *L* was asymmetrical in the individuals with amputation (Fig. 3a), typically with a greater range from the positive peak to the negative peak during the first half of the prosthetic side gait cycle in comparison to the sound side.

**Fig. 4:**
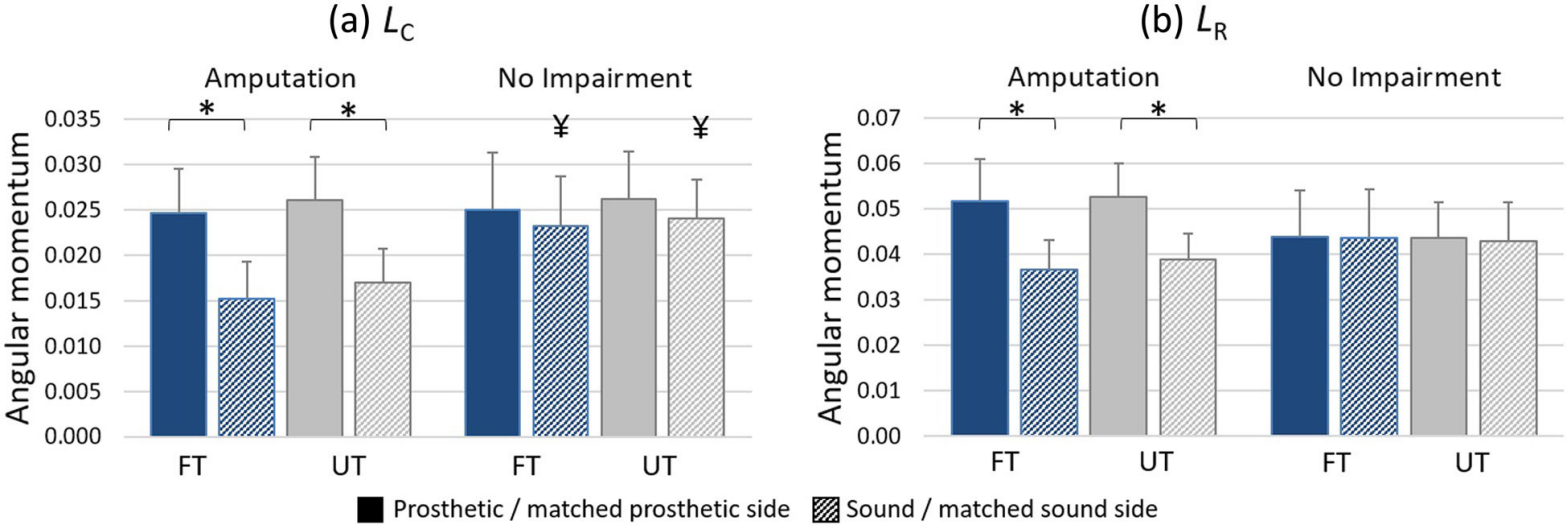
Average (mean) sagittal plane angular momentum. Values (**a**) at foot contact, (*L*_C_) and (**b**) during ipsilateral stance phase (*L*_R_), normalized to mass, height and speed (unitless). Individuals with (n = 10) and without (n = 10) amputation walking on flat (FT; blue bars) and uneven (UT; gray bars) terrain. Solid and dashed bars represent prosthetic and sound (or matched prosthetic and sound) limbs respectively. Pairwise comparisons: ‘*’ significant difference between limbs (prosthesis vs sound); ‘¥’ significant difference between groups (amputation vs no impairment); all at *p* = 0.05

Average *L*_c_ results revealed that normalized angular momentum was greater (more positive) at foot contact on UT in comparison to FT, through a main effect of terrain (F = 9.631; *p* = 0.006; η_P_^2^ = 0.345). Individuals with amputation had a greater (more positive) *L*_C_ when stepping onto the prosthetic side (*p* < 0.001) in both FT and UT conditions, but there was no inter-limb difference in the unimpaired participants (*p* = 0.161) (Fig. 4a), illustrated by a significant limb*group interaction (F = 14.065; *p* = 0.001; η_P_^2^ = 0.439).

For average *L*_R_ a group*limb interaction (F = 91.872; *p* < 0.001; η_P_^2^ = 0.836) indicated a higher angular momentum range on the prosthetic side of the amputee group (p < 0.001; Fig. 4b). There was no main effect of terrain (F = 0.494; *p* = 0.491; η_P_^2^ = 0.027).

### Variability (standard deviation) of *L*_C_ and *L*_R_

**Fig. 5:**
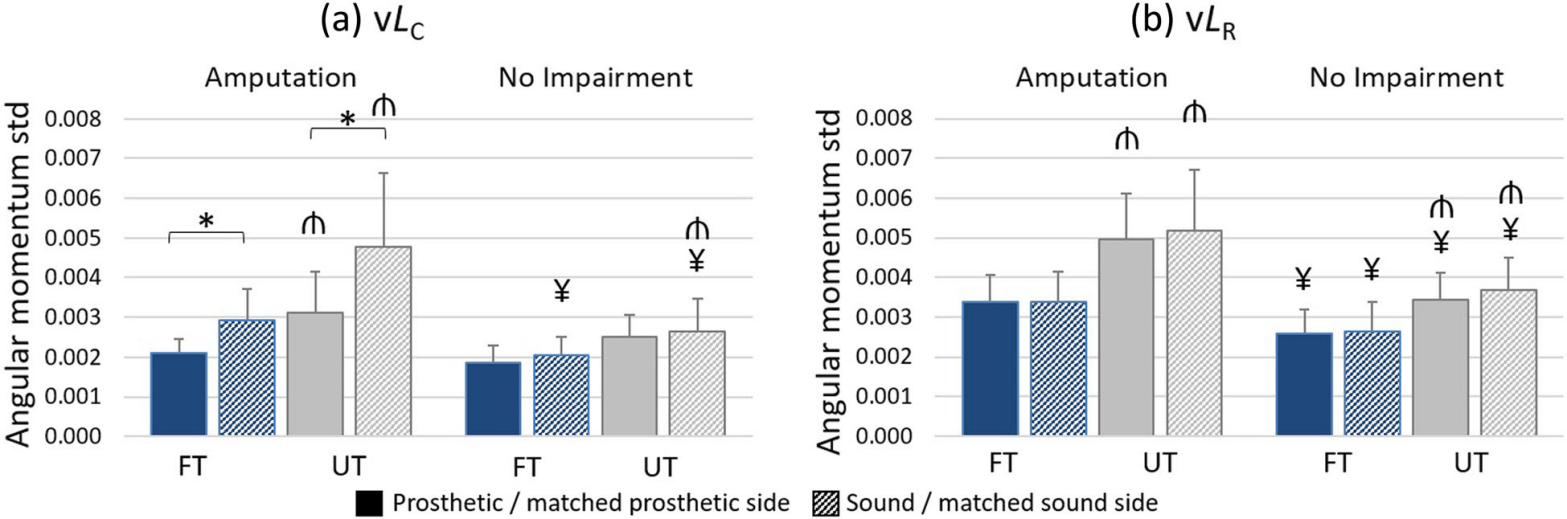
Variability (standard deviation) of sagittal plane angular momentum. Values (**a**) at foot contact (v*L*_C_) and (**b**) during ipsilateral stance phase (v*L*_R_), normalized to mass, height and speed (dimensionless). Individuals with (n = 10) and without (n = 10) amputation walking on flat (FT; blue bars) and uneven (UT; gray bars) terrain. Solid and dashed bars indicate prosthetic and sound (or matched prosthetic and sound) limbs respectively. Pairwise comparisons: ‘*’ significant difference between limbs (prosthesis vs sound); ‘¥’ significant difference between groups (amputation vs no impairment); ‘₼’ significant difference between terrain conditions (flat versus uneven); all at *p* = 0.05

The angular momentum at foot contact showed higher variability on UT than on FT in both limbs of the amputation group (*p* < 0.001), but only reached significance on the matched sound limb of the unimpaired group (matched sound – *p* = 0.09; matched prosthetic side – *p* = 0.067) (Fig. 5a) indicated by a terrain*limb*group interaction for v*L*_C_ (F = 8.765; *p* = 0.008; η_P_^2^ = 0.327).

The angular momentum range was more variable on UT in comparison to FT and more variable in the amputation group in comparison to the no impairment group, suggested by main effects of terrain (F = 49.104; *p* < 0.001; η_P_^2^ = 0.732) and of group (F = 12.458; *p* = 0.002; η_P_^2^ = 0.409) for v*L*_R_. Inter-limb comparisons were non-significant (*p* > 0.05; Fig. 5b).

## Discussion

### Sagittal plane *L* is asymmetrical in individuals with amputation (hypothesis 1)

The average results, *L*_C_ and *L*_R_, as predicted, support those from previous studies [18, 19]; a lower *L*_C_ when stepping onto the sound side and a greater *L*_R_ on average during the first 50% of the prosthetic limb stride. Similar findings have been previously attributed to a reduced propulsion capacity and braking ability of the prosthetic limb [18, 19], and this explanation holds given the results of the present study. On uneven ground, individuals with amputation retained the features seen on level ground, and an increase in *L*_C_ was observed on the UT, potentially indicating a greater demand to arrest momentum in early stance to prevent a forwards fall. The changes in *L*_C_ due to the UT appear moderate in comparison to this inter-limb disparity, however. Similarly, no difference in *L*_R_ across terrain conditions was observed. It was anticipated that the sensory and mechanical deficits of passive prosthesis use would lead to an exacerbation of the effect of uneven terrain in the amputee group, given that any profound changes in movement strategies would disrupt the balance of segmental momenta. Although *L*_C_ increased, no interaction effect reached significance, so our first hypothesis was only partially supported.

### Variability of *L* is greater in individuals with amputation on uneven terrain (hypothesis 2)

The uneven terrain surface evoked increases in the variability of angular momentum profiles in comparison to flat treadmill walking, as anticipated. In comparison to the average results, the effect of terrain on *L* variability was more apparent.

The variability of *L*_C_ and *L*_R_ was greater on UT in individuals both with and without amputation, however it was higher on average in the former group, in support of our second hypothesis. The higher variability of *L*_C_ at sound foot contact is consistent with the proposition that a reduced ability to control movement over the prosthetic side stance phase results in greater fluctuations in whole body angular momentum at the foot contact of the swing limb. This would emphasize a greater demand on the sound side for correcting movement to maintain balance whilst walking on the uneven surface. The increase in v*L*_C_ only reached significance unilaterally in the control group; a finding that may be related to limb dominance. Although it was not measured explicitly, there were more right than left ‘prosthetic sides’ which may have introduced a bias given the tendency for similarities in lateral preference amongst the general population [32]. Such a finding would corroborate previous work that exposes a difference in functional roles taken on by the two limbs during gait tasks, with respect to propulsion and control (see [33] for a review).

It was anticipated that *L*_R_ on the prosthetic side would be directly related to the contours encountered, given a reduced capacity to brake during prosthetic single limb stance, and the potential for the passive component to hinder or exaggerate shank progression. Thus, the variability would be increased further on UT on the prosthetic side in comparison to the sound side. The variability of *L*_R_ was greater on UT, however values were similar on both sides, and therefore our second hypothesis was only partially supported. As the first 50% of the stride incorporates both the single limb stance of the ipsilateral limb and an initial double support period it is possible that corrections are made by the sound side during push off to redirect the ground reaction force and attenuate potential fluctuations in *L* during prosthetic single leg stance [9]. It is also possible that this is related to differences in prosthetic step length or time. The analysis of the single and double support phases of gait in isolation may provide further insight into the relative contributions of the sound and prosthetic limbs.

Given the variability observed, it is plausible that the lack of a large change in average *L* is a result of averaging across several steps, during which the ground reaction force due to the surface profile might either act to increase or reduce angular momentum. For example, it may be only the steps that contacted a descending contour or lower level that lead to an increase in angular momentum. Pearson’s correlations, performed post-hoc to explore this further, revealed only a minority of participants with a significant relationship between the step-to-step change in surface height and *L*_C_ (further detail provided in Additional file 2). However, inconsistencies in the extent and direction of the relationship across participants indicated the lack of a uniform, definitive response.

Overall, our findings point to a greater potential for destabilization in the individuals with amputation, and a greater demand on the sound side for controlling movement. However, it is unclear to the extent to which these effects of UT may be ameliorated by appropriate intervention, either via rehabilitation or prosthetic technology. Unimpaired individuals have been shown to refine their movement on uneven terrain over a short period of time [22]. Participants in this study were only examined over a very short time period and were given limited time to familiarize with the terrain prior to testing; necessary in order to avoid excessive fatigue. It is possible that more efficient movement strategies may have been adopted with further exposure to the terrain surface. This would suggest that problems experienced on uneven ground by individuals with an amputation may be due to a lack of practice and familiarity rather than solely a deficit of the prosthesis.

This study was restricted to the assessment of individuals using passive prostheses. The provision of positive net work during stance to address the propulsion deficit of passive devices has been shown to reduce, although not completely normalize, the range of *L* on slopes [34]. Microprocessor control of powered and non-powered devices may produce more appropriate lower extremity behavior in different loading contexts and aid the user in negotiating uneven terrain (see [16] for a relevant review). It is likely, however, that the extent to which such a device will facilitate walking on uneven terrain will depend on the effectiveness of the control algorithm employed. For example, should the push-off of a powered device be inappropriately timed due to changes in contact patterns of the foot with the ground, increases in v*L*_C_ and v*L*_R_ might be observed on UT. Such findings may be of high utility for the identification of the deficits of control algorithms and potential solutions for their refinement. However, regardless of the extent to which the foot replicates biological action, there will be unpredictability introduced by the component itself if the user is not directing its motion. Electromyographic control (see [35] for an example) that increases the influence of the user on device behavior may lead to an improvement in whole-body coordination on non-level surfaces, permitting safer and more efficient walking.

There were a number of limitations to this study. It is of note that foot contact timings were based on kinematic features, specifically the relative velocities of the feet with respect to the pelvis [36]. They therefore may not have captured the true moment of interaction of the foot with the ground, affecting the *L* values extracted at heel contact. *L*_R_ and v*L*_R_, in contrast, would likely be unaffected. The potential effect of incorrectly identifying the correct contact event was explored in the 60-stride time series of two unimpaired participants. Shifts of 0, 1 and 2% of the gait cycle, corresponding to up to 2.5 frames, were introduced to the foot contact event timings with a uniform random distribution across strides. *L*_C_ and v*L*_C_ were re-calculated and compared to the original values. Differences in average *L*_C_ between the manipulated and original time series were less than 10% of the grand mean *L*_C_ and comparable across terrains, increasing confidence in our comparisons. The differences in v*L*_C_ were considerably higher; between 20 and 100% of the grand mean. When the walking surface fluctuates, however, it seems likely that the variability in foot contact timings would be under- rather than overestimated when based on kinematic patterns alone. In this case, the differences in v*L*_C_ between FT and UT would actually be greater. Nevertheless, more precise identification of contact events through the use of foot switches or accelerometers could lead to our results for v*L*_C_ being refuted.

The exclusion of the arms, hands and head from the model may have led to inaccuracies in estimation of *L*, however, the contributions of these segments to sagittal plane *L* have been shown to be negligible in comparison to those of the other segments [6]. The calculation of the inertial parameters of the lower limb of the affected side might be more influential. In the absence of geometrical information and precise center of mass values for the residual limb and prosthesis we chose to modify the inertial properties of the shank segment according to the generic correction presented by Ferris et al. [28]. A brief examination into the effect of computationally manipulating inertial values revealed that considerable differences in the range of *L* could be attributed simply to a difference in the mass and center of mass position values of the lower leg input into the model. In fact, applying no correction for the prosthesis resulted in some cases in no observable difference in *L* between the sound and prosthetic sides. It is possible that the use of more precise estimates of inertial properties through reaction board and oscillation techniques [37] could refute the results of our study, however within-limb findings would likely hold.

Along similar lines, height, mass and preferred walking speed were not matched across groups, all of which have a bearing on absolute values of angular momentum. Although normalization to these factors improves the appropriateness of comparisons across individuals and studies, the results should be interpreted with caution.

Participants were asked to maintain the hand posture they had adopted during familiarization on the uneven terrain, i.e. either no use of handrails, or light touch on handrails. More participants in the amputation group used the handrails, and three participants changed their hand position when they returned to the UT after the FT trial due to a lack of confidence. With the inclusion of hand position as a covariate, no effect of hand position was found (*p* > 0.05 for all main effects and interactions), however, suggesting that this did not confound our results. Further, the greater use of rails in the amputation group would more likely induce a reduction rather than an increase in variability, and without rails the differences between groups would be larger.

The use of handrails by the majority of participants precluded the assessment of coronal and transverse plane *L*, and medial-lateral dynamics, both of which may provide complementary insight into the deficits of the prosthetic limb and the control strategies employed in light of them. The lack of a subtalar joint, for example, may limit inversion and eversion, with implications for lateral stability [38, 39]. That most participants were unable to perform the task without handrail use is in itself of interest. Future work focused on whether the source of this inability is due to a mechanical or a perception deficit is warranted.

## Conclusion

The less effective regulation of *L* in individuals with amputation in comparison to unimpaired individuals appears to be challenged further on uneven ground. Our results point to a greater onus being placed on the sound limb to control movement and regulate *L* in light of the changing surface underfoot, due to the deficiencies of a passive prosthesis. The employment of an intervention paradigm during which individuals are exposed progressively to uneven terrain may determine whether it is possible for individuals with amputation to arrive at more efficient movement solutions in light of these deficits.

## Additional files

Additional file 1: Full Body Model: Detailed description of the marker placement protocol and biomechanical model applied. (DOCX 36 kb)

Additional file 2: Relationship between surface profile and whole body angular momentum: Details of post hoc analysis performed to explore the potential relationship between surface profile and angular momentum at foot contact. (DOCX 154 kb)

## Abbreviations

FT: Flat terrain
*L*: Whole body angular momentum
*L*_C_: Average *L* at foot contact
*L*_R_: Average range of *L* in the first 50% of the gait cycle
UT: Uneven terrain
v*L*_C_: Variability of *L* at foot contact
v*L*_R_: Variability of the range of *L* in the first 50% of the gait cycle
