# Supplementary material for: Uneven terrain exacerbates the deficits of a passive prosthesis in the regulation of whole body angular momentum in individuals with a unilateral transtibial amputation
Source: J Neuroeng Rehabil. 2019 Feb 4;16:25. doi: 10.1186/s12984-019-0497-9 (PMC6360756; doi:10.1186/s12984-019-0497-9)
Supplement: Supplementary file 1 — Full Body Model: Detailed description of the marker placement protocol and biomechanical model applied. (DOCX 36 kb) [file 12984_2019_497_MOESM1_ESM.docx]

FULL BODY MODEL – DESCRIPTION

# Model Fundamentals

| **Design** | In house developed lower limb, pelvis, trunk and hand model, and location protocol. |
| --- | --- |
| **Degrees of freedom** | All joints/segments : 6 |
| **Optimisation** | Segment optimisation - Software generic.  See Visual 3D documentation at  *http://www.c-motion.com/v3dwiki/index.php?title=Six_Degrees_of_Freedom* and 1 |
| **Kinetic calculations** | Mass, moments of inertia and center of gravity calculated according to ^2^ (see Visual 3D documentation at *http://c-motion.com/v3dwiki/index.php/Segment_Geometry* |

# Landmarks

| **Landmark ID**  **(^bilateral/side)** | **Location** | **Location method** | **Function** | | **Type** |
| --- | --- | --- | --- | --- | --- |
|  |  |  | Definition | Tracking |  |
| ^ASI | Superficial on skin surface such that marker body is anterior to prominent anterior edge of iliac crest. | Palpation | Yes | Yes | Marker |
| ^PSI | Superficial to palpable prominence at posterior edge of iliac crest. | Palpation | No | Yes | Marker |
| ^VPSI | PSI projected by half marker radius perpendicular to contralateral PSI in plane connecting PSIs and mid point between ASIs. | Calculation | Yes | No | Derived |
| ^VASI | ASI projected by half marker radius perpendicular to contralateral ASI in plane connecting ASIs and mid point between PSIs. | Calculation | Yes | No | Derived |
| ^HJC | Hip joint centre – at the following distances from mid-point between VASI markers (Pelvis origin) in pelvis coordinate system (see Pelvis):  AP (in mm) = -0.24PD - 9.9  ML (in mm) = 0.28PD + 0.16PW +7.9  Axial (in mm) = -0.16PW-0.04LL-7.1  Where PD = Pelvic depth: the distance between the mid points of VASIs and VPSIs  PW = Pelvic width : distance between left and right VASIs  LL = Leg Length : distance between the ASI and VANM via the VKNM ^3^ | Calculation | Yes | No | Derived |
| ^TRO | Lateral and superficial to the centre of the palpated prominence of the greater trochanter when standing in calibration posture. | Palpation | No | Yes | Marker |
| ^TH1 | Anteriorly on thigh, approximately 1/3 distance between hip and knee. | Visualisation | No | Yes | Marker |
| ^TH2 | Anteriorly on thigh, 50mm (approx.) above the patella when relaxed in standing. | Visualisation | No | Yes | Marker |
| ^TH3 | Laterally on thigh, approximately mid distance between hip and knee | Visualisation | No | Yes | Marker |
| ^KNL | At the bony prominence of the lateral femoral condyle, to form the lateral end of a ‘knee axis’ with ^KNM, superficially on the prosthetic socket where applicable. | Palpation | Yes | Yes | Marker |
| ^KNM | At the bony prominence of the medial femoral condyle, to form the medial end of a ‘knee axis’ with ^KNL, superficially on the prosthetic socket where applicable. | Palpation | Yes | No | Marker* |
| ^VKNL | KNL projected by half marker radius in direction of KNM. | Calculation | Yes | No | Derived |
| ^VKNM | KNM projected by half marker radius in direction of KNL. | Calculation | Yes | No | Derived |
| ^KJC | Mid point between KNL and KNM. | Calculation | Yes | No | Derived |
| ^SK1-4 | Set of four markers placed anteriorly and posteriorly on lower 1/3 of the shank positioned to avoid excessive rotation due to individual tendon/muscle protrusion on dorsi/plantar flexion. | Visualisation | No | Yes | Marker |
| ^ANL | At most lateral point on lateral malleolus, to form the lateral end of the ‘ankle’ axis with ^ANM, or on the border of the prosthetic foot shell, aligned laterally with the base of the prosthetic pylon. | Palpation | Yes | No | Marker |
| ^ANM | At most medial point on medial malleolus, or on the border of the prosthetic foot shell, aligned medially with the base of the prosthetic pylon, to form the medial end of the ‘ankle’ axis with ^ANL. | Palpation | Yes | No | Marker^*^ |
| ^VANL | ANL projected by half marker radius in direction of ANM. | Calculation | Yes | No | Derived |
| ^VANM | ANM projected by half marker radius in direction of ANL. | Calculation | Yes | No | Derived |
| ^AJC | Mid point between ANL and ANM |  | Yes | No | Derived |
| ^TOE | At a point approximating position of second metatarsal head on dorsum of shoe. Prosthetic side, where applicable, matched to the position on the shoe of the unaffected side. | Palpation /Visualisation | Yes | Yes | Marker |
| ^MT1 | On dorsum of shoe at a point approximating position of first metatarsal head. Prosthetic side, where applicable, matched to the position on the shoe of the unaffected side. | Palpation /Visualisation | Yes | No | Marker^*^ |
| ^MT5 | On dorsum of shoe at a point approximating position of fifth metatarsal head. Prosthetic side, where applicable, matched to the position on the shoe of the unaffected side. | Palpation /Visualisation | Yes | Yes | Marker |
| ^LHL | Laterally on heel counter, below ^ANL, approximately 15mm from the ground. | Palpation /Visualisation | No | Yes | Marker |
| ^HEE | On heel counter, approximately 15mm from ground, centrally when viewing from a posterior position along the long axis of the shoe to form the longitudinal axis of the foot with TOE | Measurement /Visualisation | Yes | Yes | Marker |
| STRN | Anterior and superficial to sternal notch | Palpation | Yes | Yes | Marker |
| XYPH | Superficial to xyphoid process | Palpation | Yes | Yes | Marker |
| C7 | Superficial to seventh cervical vertebra | Palpation | Yes | Yes | Marker |
| LUM | Lumbar region, superficial to the spine at point of maximum curvature. | Palpation | Yes | Yes | Marker |
| LowerTorso | Mid point between XYPH and LUM | Palpation | Yes | No | Marker |
| UpperTorso | Mid point between STRN and C7 | Palpation | Yes | No | Marker |
| ^HA1 | Between 1^st^ and 2^nd^ metacarpals, approximately 15mm from the metacarpal heads. | Palpation | Yes | Yes | Marker |
| ^HA2 | On the radial process of the wrist, to form a ‘wrist axis’ through the joint with HA3. | Palpation | Yes | Yes | Marker |
| ^HA3 | On the ulnar process of the wrist, to form a ‘wrist axis’ through the joint with HA2. | Palpation | Yes | Yes | Marker |
| ^FIN | HA1 projected distally 0.5*distance between HA1 and midpoint of HA2 and HA3, along the line formed by HA1 and then midpoint of HA2 and HA3 | Calculation | Yes | No | Derived |

* Marker removed following static calibration trial

# Segment definitions

| **Segment (^bilateral/**  **side)** | **Landmarks** (derived landmarks in parentheses) | **Origin** | **Axes** | | | **Geometry***  **Joint radii** | **Tracking markers** |
| --- | --- | --- | --- | --- | --- | --- | --- |
|  |  |  | **Flex/ext** | **Add/abd** | **Axial** |  |  |
| Pelvis (V3D Composite^a^) | LASI, RASI, LPSI, RPSI, (LVASI), (RVASI), (LVPSI), (RVPSI) | Midpoint between LVASI and RVASI markers | Parallel to line from origin to RVASI | Orthogonal to the flex/ext and axial axes. | Perpendicular to the plane defined by LVASI, RVASI & the midpoint between LVPSI and RVPSI | See ^a^ | LASI, RASI, LPSI, RPSI, VSAC |
| ^ Thigh | (VKNL), (VKNM), TRO, TH1, TH2, TH3, (HJC) | HJC | Perpendicular to axial axis in plane defined by HJC, VKNL and VKNM | Orthogonal to axial and flex/ext axes | Line joining HJC and midpoint between VKNL and VKNM | Proximal: half distance between RTRO and LTRO | TRO, TH1-3 |
| ^ Shank | (KJC), (VANL), (VANM), SK1-4 | KJC | Perpendicular to axial axis in plane defined by KJC, VANL and VANM | Orthogonal to Axial and Flex/Ext axes | Line joining KJC and midpoint between VANL and VANM | Proximal: half distance between VANL and VANM | SK1-4 |
| ^ Foot | (AJC), (VANL), TOE, LHL, HEE, MT1, MT5 | AJC | Perpendicular to axial axis in plane defined by AJC, VANL and TOE | Orthogonal to Axial and Flex/Ext axes | Line joining AJC and TOE | Proximal : distance between AJC and VANL, Distal : half distance between MT1 and MT5 | TOE, HEE, MT5, LHL |
| Thorax | (UpperTorso), (LowerTorso), STRN, XYPH, C7, LUM. | Upper Torso | Perpendicular to axial axis, orthogonal to plane defined by UpperTorso, LowerTorso and STRN. | Orthogonal to Axial and Flex/Ext axes | Line joining UpperTorso and LowerTorso | Proximal : half distance between RASI and LASI  Distal : half distance between RTRO and LTRO | STRN, XYPH, C7, LUM |
| Hand^c^ | (FIN), HA1, HA2, HA3 | Midpoint between HA2 and HA3 | Perpendicular to axial axis in plane defined by FIN, HA2 and HA3 | Orthogonal to Axial and Flex/Ext axes | Line joining FIN and midpoint between HA2 and HA3 | Distal : half distance between HA2 and HA3 | HA1, HA2, HA3 |

^a^ See <http://www.c-motion.com/v3dwiki/index.php?title=V3D_Composite_Pelvis>

^b^ Not used for kinematic analysis in current study.

# Joint definitions / rotation sequences

| **Joint / Segment angle name (*bilateral)** | **Segment^a^** | **Reference segment^a^** | **Positive direction (sagittal plane)^b^** |
| --- | --- | --- | --- |
| Pelvis | Pelvis | Laboratory | Tilt – anterior |
| Hip | Thigh | Pelvis | Flexion |
| Knee | Shank | Thigh | Flexion |
| Ankle | Foot | Shank | Dorsiflexion |

^a^ Angles are calculated within the co-ordinate system of the reference segment.

^b^ Sagittal plane first in Cardan sequence for all joints.

# System

| **Capture system / software** | Motion Analysis Corporation ^a^: 12-camera Raptor/ Cortex version 6 ^a^ |
| --- | --- |
| **Medium** | Passive retro-reflective markers : 12.7mm with thin fabric base |
| **Sampling frequency** | 100Hz |

# Processing

|  | **Details** | **Software** |
| --- | --- | --- |
| **Eventing** | Kinematic algorithm (velocity-based, from 4) | Visual 3D^b^ |
| **Filtering** | 7Hz 4^th^ order Butterworth, by marker ^5^ | Visual 3D^b^ |
| **Interpolation** | Cubic spline / software-based virtual join ^a^ | Cortex ^a^ |

^a^ Motion Analysis Corporation, Santa Rosa, CA, USA

^b^ C-Motion, Germantown, MD, USA

^c^ Mathworks, Natick, MA, USA

# References

^1^ Spoor CW. Rigid body motion calculated from spatial co-ordinates of markers. J. Biomech 1980; 13: 391-393.

^2^ Hanavan Jr, EP. A mathematical model of the human body (No. AFIT-GA-PHYS-64-3). Air Force Aerospace Medical Research Lab Wright-Patterson Afb Oh, 1964.

^3^ Harrington ME, Zavatsky AB, Lawson SE, Yuan Z, Theologis TN. Prediction of the hip joint centre in adults, children, and patients with cerebral palsy based on magnetic resonance imaging. J Biomech. 2007;40(3):595-602.

^4^ Zeni Jr, JA., Richards, JG, & Higginson, JS. Two simple methods for determining gait events during treadmill and overground walking using kinematic data. Gait Posture 2008, 27(4), 710-714.

^5^ Giakas, G.. Power spectrum analysis and filtering. Innovative Analyses of Human Movement, Champaign, IL: Human Kinetics, 2004.
