# Supplementary material for: Uneven terrain exacerbates the deficits of a passive prosthesis in the regulation of whole body angular momentum in individuals with a unilateral transtibial amputation
Source: J Neuroeng Rehabil. 2019 Feb 4;16:25. doi: 10.1186/s12984-019-0497-9 (PMC6360756; doi:10.1186/s12984-019-0497-9)
Supplement: Supplementary file 2 — Relationship between surface profile and whole body angular momentum: Details of post hoc analysis performed to explore the potential relationship between surface profile and angular momentum at foot contact. (DOCX 154 kb) [file 12984_2019_497_MOESM2_ESM.docx]

**Is there a relationship between surface profile and angular momentum at foot contact, *L*_C_?**

*Background*

This post hoc analysis was performed in order to explore whether there was a relationship between the change in step height and the whole body angular momentum at the time of foot contact (*L*_C_) following, while walking on uneven terrain. It was hypothesized that a strong relationship would be observed at sound limb foot contact due to the lack of ability to discern and correct for the profile of the surface during prosthetic limb stance.

*Methods*

Data from ten individuals with amputation and ten age- and gender- matched control participants were analyzed. To characterize the surface profile, the height of the ankle joint center at the instance at which the toe marker of the contralateral (swing) limb passed the stance limb heel marker was extracted as a measure of step height, and differenced to approximate changes in surface height from step to step.

Post hoc Pearson's correlations were performed to determine whether a relationship existed between *L*_C_ and the change in surface height from the preceding step, with strength of correlation assessed according to Cohen’s criteria; r > 0.5, 0.3 and 0.1, for strong, moderate and weak correlations respectively [1].

*Results and Discussion*

Only half of the amputee participants revealed significant correlations between step height and subsequent *L*_C_ on the prosthetic side (strong n=2; medium n=3) and two of ten on the sound side (strong n=1; medium n=1; see Figure S2-1 for exemplary data) (Figure S2-2a). In contrast, only two participants in the no impairment group revealed correlations between *L*_C_ and preceding step height on either limb; one strong and one medium (Figure S2-2b).

This correlational post hoc analysis revealed that few participants showed a significant relationship between *L*_C_ and the change in step height from the preceding step and it would appear that a greater number of occurrences were observed in stepping onto the prosthetic limb rather than the sound limb, refuting our hypothesis. The significant relationships were not in a uniform direction, i.e. negative or positive, suggesting that there is not a consistent strategy employed across individuals. It is of note that the analysis applied explored instantaneous surface height but not contour, which may have acted to slow or propel shank movement, affecting subsequent *L*_C_.


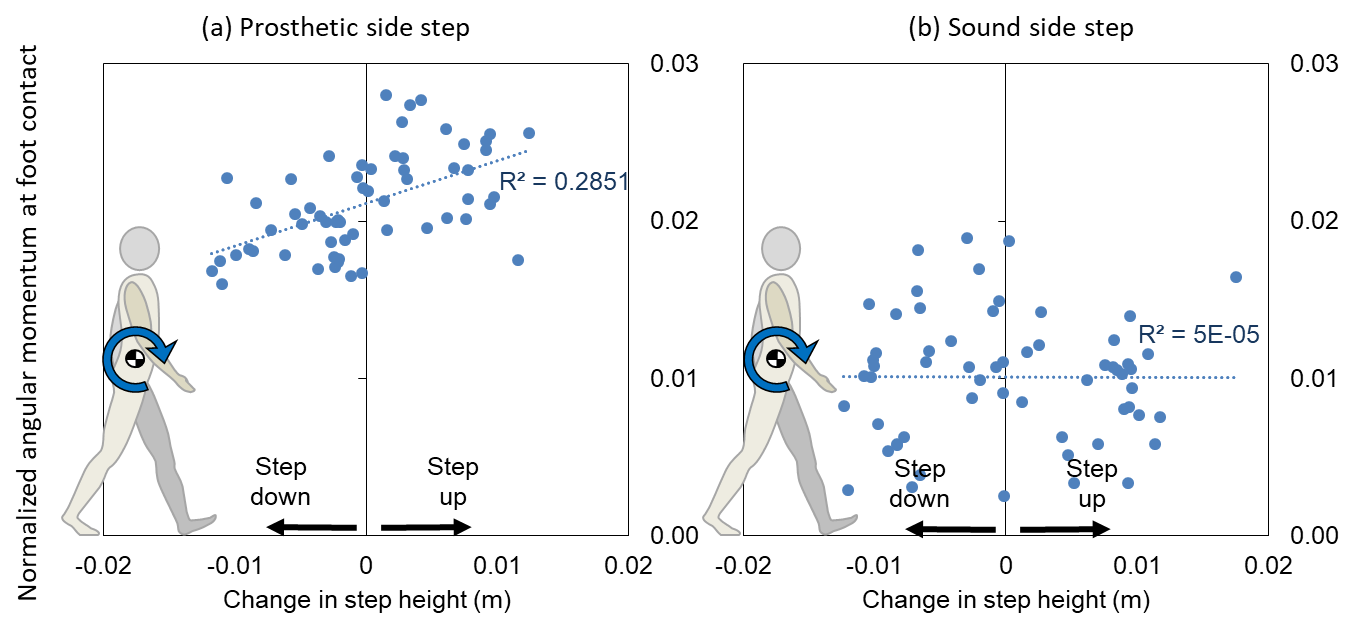


**Figure S2-1. Relationship between angular momentum and previous step height.** Correlations between angular momentum at foot contact and change in height from previous step. Data from one 65 yr old male with a right transtibial amputation walking on uneven terrain. A significant moderate correlation is observed on the prosthetic side only (a), indicating that stepping onto a higher level results in a greater positive angular momentum at heel contact of the prosthetic side. There is no such association on the sound side (b). This was not observed uniformly across participants.


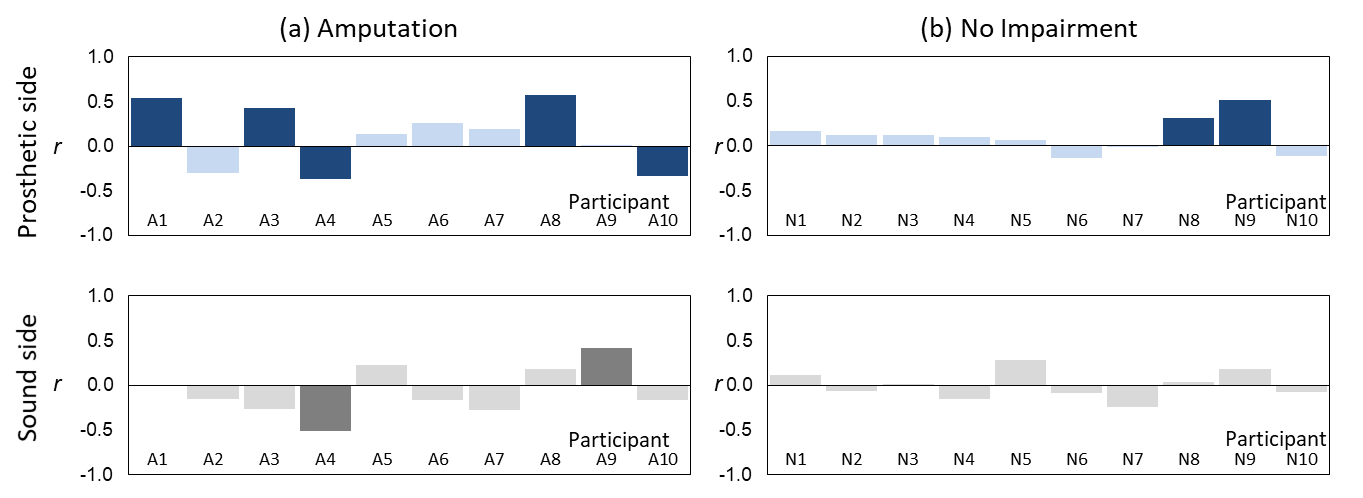


**Figure S2-2.** **Pearson correlations between angular momentum at foot contact and preceding step height difference.** Individuals with (a) unilateral transtibial amputation (n=10; A1-10) and (b) no impairment (age and gender matched; n=10; N1-10). Prosthetic (/matched) side above; sound (/matched) side below. Dark bars indicate significant correlations with r > 0.3 (p < 0.05).

[1] Cohen J. A Power Primer. Psychol Bull. 1992;112:155-159; doi:10.1037/0033-2909.112.1.155.
